# Neural digital twins: reconstructing complex medical environments for spatial planning in virtual reality

**DOI:** 10.1007/s11548-024-03143-w

**Published:** 2024-05-06

**Authors:** Constantin Kleinbeck, Han Zhang, Benjamin D. Killeen, Daniel Roth, Mathias Unberath

**Affiliations:** 1 TUM School of Medicine and Health, Department Clinical Medicine, Orthopedics, Technical University of Munich, Munich, Germany; 2 Johns Hopkins University, Baltimore, MD USA; 3 Friedrich-Alexander-Universität, Erlangen, Germany

**Keywords:** NeRF, Robotic surgery, Machine learning, Mixed reality, Human–computer interaction

## Abstract

**Purpose:**

Specialized robotic and surgical tools are increasing the complexity of operating rooms (ORs), requiring elaborate preparation especially when techniques or devices are to be used for the first time. Spatial planning can improve efficiency and identify procedural obstacles ahead of time, but real ORs offer little availability to optimize space utilization. Methods for creating reconstructions of physical setups, i.e., digital twins, are needed to enable immersive spatial planning of such complex environments in virtual reality.

**Methods:**

We present a neural rendering-based method to create immersive digital twins of complex medical environments and devices from casual video capture that enables spatial planning of surgical scenarios. To evaluate our approach we recreate two operating rooms and ten objects through neural reconstruction, then conduct a user study with 21 graduate students carrying out planning tasks in the resulting virtual environment. We analyze task load, presence, perceived utility, plus exploration and interaction behavior compared to low visual complexity versions of the same environments.

**Results:**

Results show significantly increased perceived utility and presence using the neural reconstruction-based environments, combined with higher perceived workload and exploratory behavior. There’s no significant difference in interactivity.

**Conclusion:**

We explore the feasibility of using modern reconstruction techniques to create digital twins of complex medical environments and objects. Without requiring expert knowledge or specialized hardware, users can create, explore and interact with objects in virtual environments. Results indicate benefits like high perceived utility while being technically approachable, which may indicate promise of this approach for spatial planning and beyond.

**Supplementary Information:**

The online version contains supplementary material available at 10.1007/s11548-024-03143-w.

## Introduction

The increasing number of machines, robots, and tools in the modern operating room (OR) due to technological advancements and specialization poses a challenge for space management and complexity reduction [1]. Planning the required tools and their positioning for novel procedures in advance is essential for streamlining processes, enhancing patient safety, operating efficiency, and communication [2]. This becomes even more difficult for first time, specialized, rare, or unique procedures that have limited prior knowledge and little room for trial and error [3]. Therefore, fast and simple decision-making is crucial, as needs can change rapidly and unpredictably [4].

However, the current situation is problematic, as planning the space requirements and layout of the needed tools and machinery can be prohibitively difficult and costly. The targeted spaces are often unavailable due to the high demand and cost of ORs, and because planners or the machinery might still be remote at the time of planning [5]. Most planning happens in two dimensions on computer screens, with approaches using low-fidelity or artist-created 3D or virtual reality (VR) visualizations just becoming available. Hence, methods are needed to enable virtual spatial planning and exploration in a realistic scenario, with the relevant objects. Such VR environments can work in remote setups and do not require access to the facilities. Said methods would help anticipate and address potential needs and problems in advance, streamlining the implementation. Reducing the need for layout revisions and equipment re-positioning can in turn help to reduce procedure times, lowering cost and chances for complications.

Current software solutions for this situation, while helpful, have significant limitations. They are usually costly and labor-intensive [3], making them hard to adapt or extend with new needs or machines, especially for novices. Creating digital assets of rooms and tools is also expensive and complex. LiDAR scanners and photogrammetry are precise, but require expertise and post-processing [6]. SLAM-like methods are fast to create output and straightforward to use, but produce noisy and low-quality output [7]. Neural radiance fields (NeRFs) can generate realistic images, but their spatial reconstruction performance can be inadequate, and they have poor compatibility with existing rendering pipelines and exhibit high computational demand [8]. In addition, the suitability of state-of-the-art model output as VR environments is not yet well understood.

**Fig. 1:**
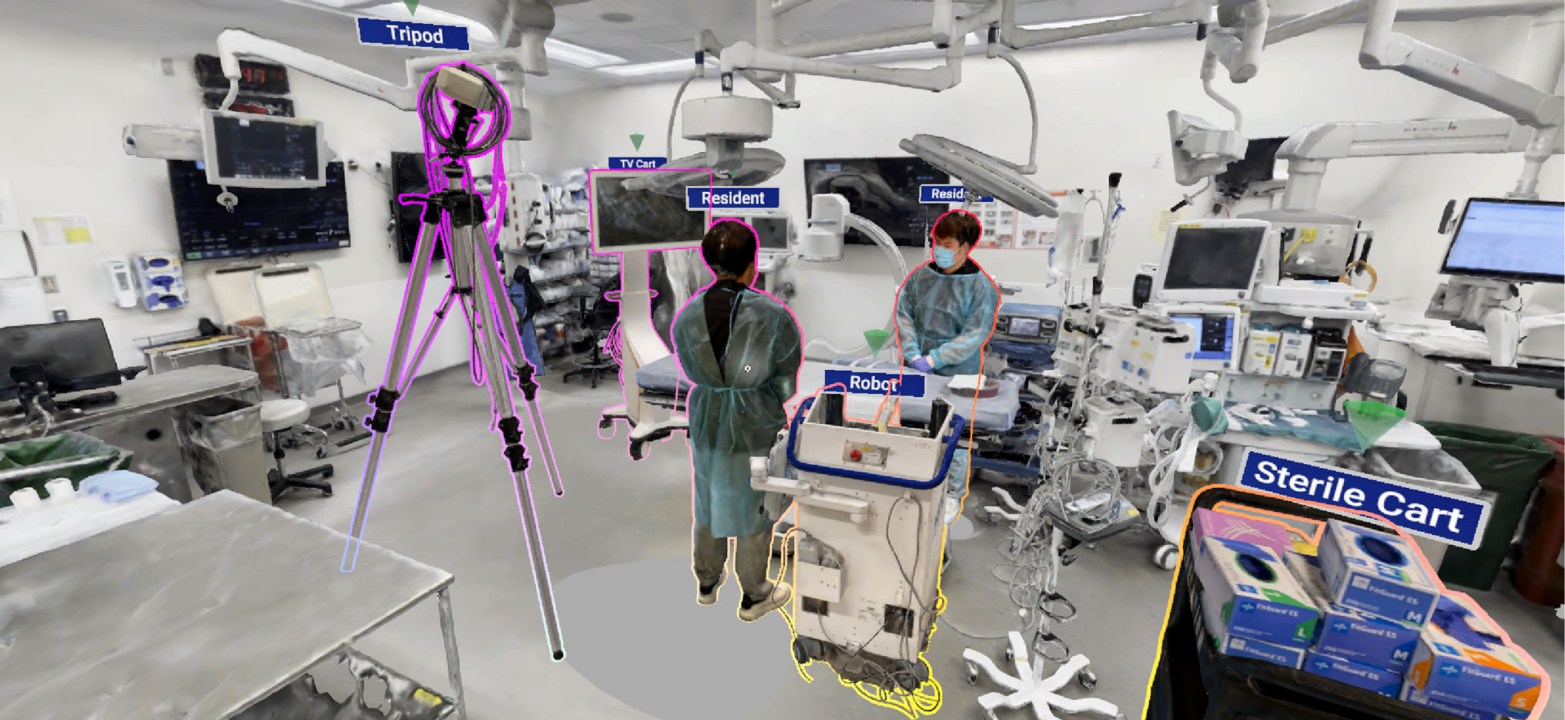
Reconstructed operating room with objects in the virtual environment

Our approach uses neural surface reconstruction of casual video captures to create digital twins of complex medical environments, such as ORs and objects, as displayed in Fig. 1. We export these reconstructions as textured polygon meshes, compatible with existing rendering pipelines and various downstream applications. Our process is mostly automated, minimizing the need for specialized 3D capture hardware and post-processing. To display these digital twins, we devised a VR environment, accessible through standalone headsets. This environment allows users to explore, as well as interact with and manipulate objects, helping them plan upcoming procedures. To evaluate our approach, we ran a user study. Participants were tasked with adjusting the layout of virtual ORs, using two different reconstructions styles: neural reconstruction and whiteboxed reconstruction. We collected both subjective and objective data, revealing significantly increased perceived utility and presence using the neural reconstruction-based environments, combined with higher perceived workload and exploratory behavior.

Our focus in this work is to evaluate whether neural reconstruction techniques would be useful and potentially even beneficial in a practical scenario in which it might be used realistically. We evaluate this utilizing a mostly automatic way to create the visualizations, with clinical planning as an example scenario. The key contribution of our work include:

1. A framework for creating digital twins of complex medical environments and objects using neural reconstruction of casual video capture.
2. An evaluation of a state-of-the-art neural reconstruction techniques for creating accurate digital twins of OR settings.
3. Preliminary insights into the cognitive impact of operating room planning within neural reconstructions compared to whiteboxed environments in VR, focusing on aspects such as workload, sense of presence, and perceived utility through a user study with non-expert participants.
4. An analysis of objective factors including user exploration and interaction patterns within these virtual environments.

The remainder of this work is organized as follows: The next section reviews the most important *Related Works*. Our reconstruction approach, rooms and objects are explained in the *Methods* section. The *Evaluation* section describes the application, tasks, measures, study population and procedure. Subjective and objective measure outcomes are presented with detailed statistics in the *Results* section. The *Discussion* section interprets the main findings and ends with limitations and a conclusion.

## Related work

Spatial planning within ORs has become increasingly complex with the influx of devices and tools. Current practices predominantly rely on the graph theoretic approach for facilities layout planning [9]. However, these methods are reaching their limitations in terms of flexibility and precision, struggling to adapt to the dynamics of modern OR environments, with the design quality a key determining factor for outcomes [10]. In parallel, there has been an increasing adoption of using VR for spatial planning, where initial findings suggest that high fidelity in VR environments can enhance spatial understanding and decision-making [11].

To overcome the challenges of spatial representation and the shortcomings of traditional modeling methods, there is a growing trend toward incorporating various data acquisition techniques. The integration of point cloud data with CAD models represents an innovative approach to streamline the modeling process, maintaining accuracy while increasing efficiency [12]. Recently, NeRFs have increasingly gained traction as a way to create arbitrary view renderings of a reconstructed scene from monocular RGB [8], with ongoing research into optimizing accuracy and speed [13], enabling applications. Relying on NeRFs directly as an environment representation is challenging, as they have demanding runtime compute requirements and do not explicitly define surfaces. Clear surfaces however are important for collision handling, enabling interaction. Further, the scene geometry is usually modeled by a density neural network. This can make it challenging to export a defined 3D surface to use in other rendering applications, as density thresholding is scene specific and does not always yield the desired results. Neural surface reconstruction methods that combine neural rendering ideas with explicit surface reconstruction can provide high-fidelity, detailed 3D models because they rely on optimizing a signed distance field [14] instead of a density approximator. These methods can be adapted to offer a robust and portable solution for reconstruction of complex environments for digital twin-based scenario modeling and planning, providing a practical solution to visualize and navigate ORs.

## Methods

### Digital twin creation

We created virtual analogs of two operating rooms and ten objects using a custom implementation of Neuralangelo [14]. We used the suggested parameters, except for reducing the hash map size to $2^{21}$ for objects. Our creation pipeline was as follows: Take a video of the target object or room using a smartphone, prepare the data and train the network, extract the mesh, cut off excess geometry, decimate it and create a mapped texture. Training took around 40–50 h using a NVIDIA Quadro RTX 6000 (see“Appendix C”). Further acceleration of the reconstruction process by factors of $>50$ is possible [13]. Data collection happened with off-the-shelf hardware (recent model iPhone). No beautification of the mesh besides cutting off superfluous parts to ensure stable performance and quality was done. Outcomes represent the state of the art in realistic neural reconstruction. Examples of the expected visual quality can be found in [14]. While our method excels at reconstructing shapes and fine details from limited source material, reflective and uniformly colored surfaces can be challenging. The capabilities of the rendering hardware represent a further limitation of the achievable quality. This procedure was chosen to require no expensive hardware, and little expert time and knowledge. The few manual steps could possibly be automated.

#### Rooms

**Fig. 2:**
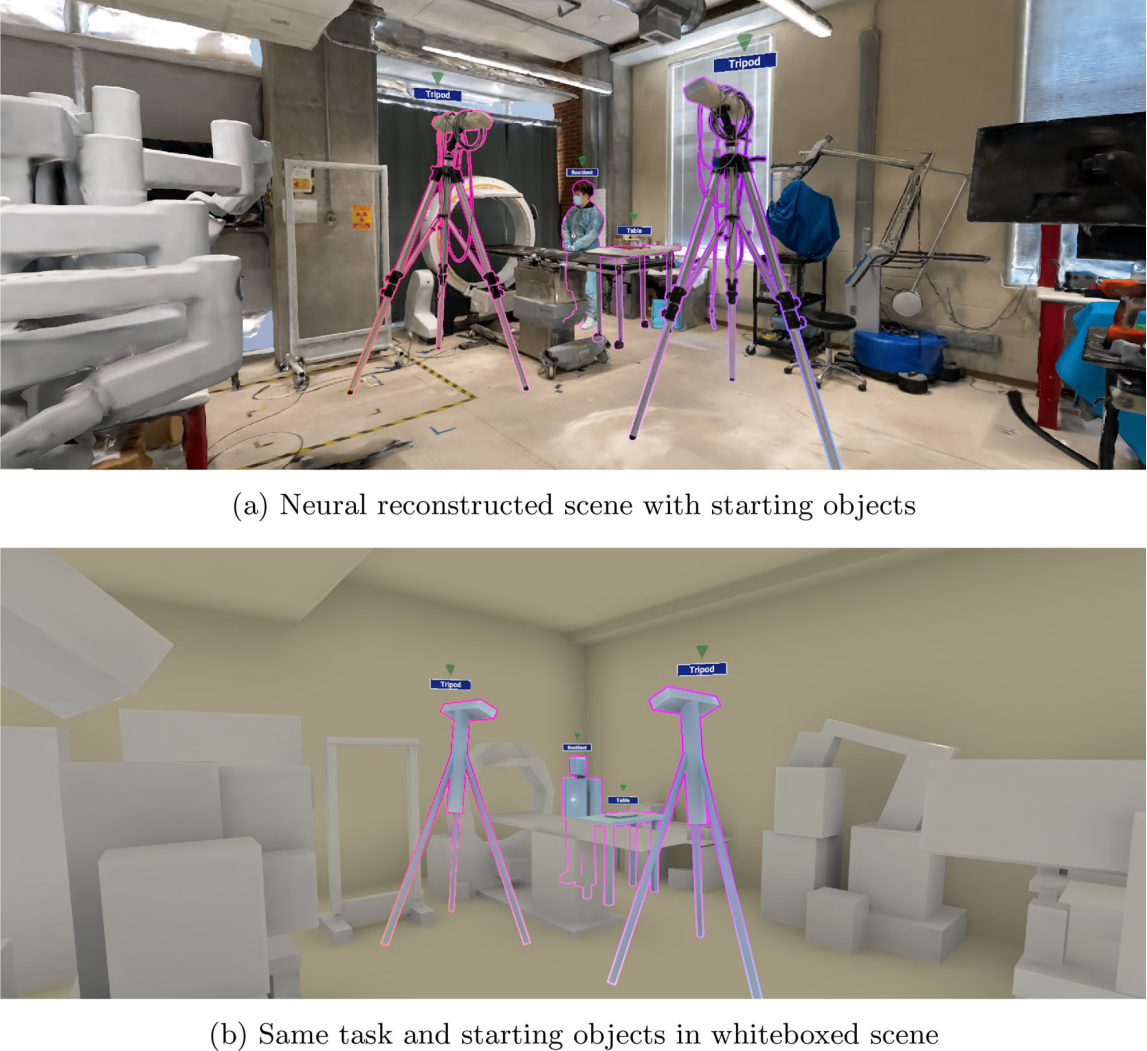
Starting objects in neural and whiteboxed reconstructions

We reconstructed two environments: a mock OR mainly used for experiments on novel robotic systems, and a real OR in daily use in a hospital. The rooms were reconstructed from around 20–25 min of wide-angle video each, depending on the complexity and details. To optimize the visuals for VR hardware, we split the meshes into two parts of around 400k faces, each with a 4k texture. This improved the visual quality and reduced the creation time and hardware requirements. Impressions from the reconstructions of the real and mock OR are shown in Figs. 1 and 2a, respectively.

#### Objects

**Fig. 3:**
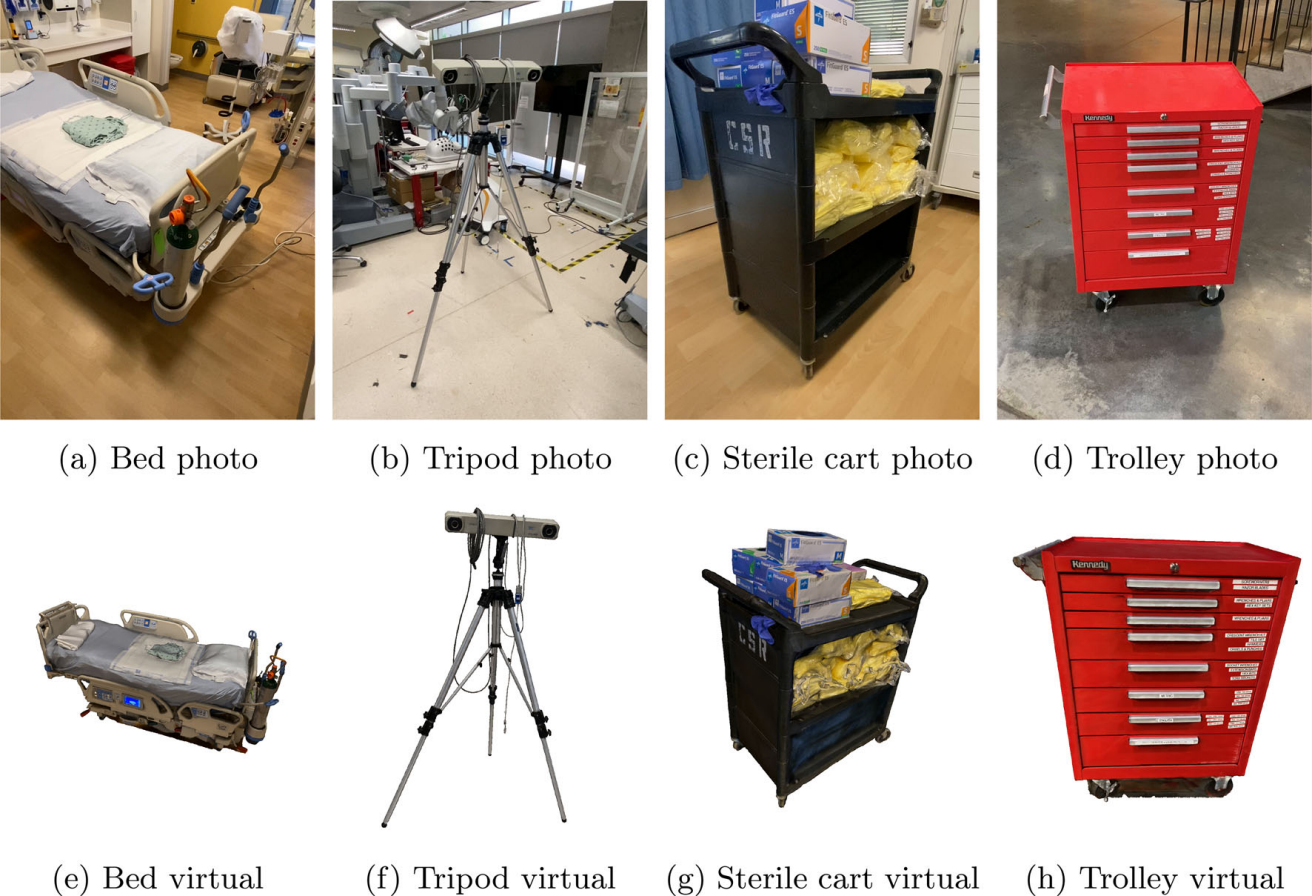
Sample photograph out of the training data for our reconstructed objects with the corresponding reconstruction below

We further created ten different reconstructions of objects found in or around the rooms used in the experiment: A *hospital bed*, *rolling chair*, *surgical robot*, *roll-able table with equipment*, *surgeon lookalike*, *trolley with medical material*, *roll-able tracking system*, *tracking system on tripod*, *workshop trolley*, and a *roll-able digital monitor*. The objects were reconstructed from around 2–4 min of video. Mesh decimation depended on the object complexity, objects had 20k to 60k faces and a 2k texture. Samples of input images and resulting models can be seen in Fig. 3.

## Evaluation

We performed a within-subject repeated measures experiment with the level of *reconstruction type*, i.e., neural reconstruction and whiteboxed reconstruction, as well as the *type of environment*, i.e., real and mock OR, as independent factors. Thus, participants evaluated two reconstruction types in two environments.

### Virtual reality application

The VR application was developed with Unity 2022.3.7 due to ease of development and deploying to VR headsets. We created whiteboxed versions of the environments and objects as a second reconstruction level for the experimental application, see Fig. 2. The whiteboxed versions were based on the neural reconstructions and represent the quality level one could realistically achieve using CAD modeling software in reasonable time without resorting to specialized hardware or relying on experts to truthfully model the environment. In addition to the 2 rooms and 10 items, the applications contain a training scene, shown before the actual experiment starts. Users can look around and move freely in the virtual environment, physically within around $1~\hbox {m}^{2}$, virtually additionally via teleportation. It is possible to create, move (near and far interaction, one- and two-handed), and delete objects. The VR application mirrors the user and objects positions onto the supervisor’s laptop. The system was developed to run locally on a Meta Quest 2 head mounted display (HMD) device, with using controllers to allow user input. In the experimental setup, we use a secondary laptop to let the study supervisor issue commands and load environments remotely on the headset. Communication between the laptop and HMD was wireless via Wi-Fi.

### Tasks

To engage participants in the virtual environment, they were tasked to do a simple planning task for the OR. They had to place, move and remove objects while considering the objective at hand. The instructions were displayed next to the application menu. Included were information about target objects, which constraints to be applied, and the objectives. While the task were simple enough to ensure approachability within the VR exposure time, the tasks were inspired by realistic planning scenarios. Additionally, some objects were already placed in the scene, exemplary as a current setup. These could be moved or removed. We designed the tasks to encourage participants to experience the room and objects, and stay engaged during the VR trials for comparable exposure. Therefore, we did not specify the tasks too much to enable exploration. Actual planning outcomes were not quantified. See “Appendix A” for examples.

### Measures

#### Perceived utility

A custom scale was used to assess the perceived utility of the application. It consisted of eight questions with the three subscales *confidence*, *memorability*, and *utility*. All questions were to be answered on a 7-point Likert scale with endpoints *fully disagree (1)* and *fully agree (7)*. The questions can be found in “Appendix B.”

#### Presence

We used the Igroup Presence Questionnaire (IPQ) by Schubert et al. [15] to measure presence in the virtual environment. It consists of 14 items on four subscales (general, spatial presence, involvement, perceived realism) on a 7-point Likert scale with varying endpoints.

#### Perceived task load

To assess workload and performance the NASA Task load index (NASA-TLX) [16] was used, including all subscales (mental, physical, and temporal demand and performance, effort, and frustration), without pairwise comparisons (raw TLX).

#### Motion sickness

The Fast Motion Sickness Scale (FMSS) by Keshavarz et al. [17] was used to check whether participants were negatively affected by the experience using one question. Scale width is 0 (no sickness) to 100 (very high sickness).

Lastly, we asked participants to rate their familiarity with the presented virtual environment on a 7-point Likert scale. The endpoints of the scale were *completely unknown (1)* and *very familiar (7)*.

We further evaluate participants’ behavior inside the virtual environment as objective measures. We log the following regarding locomotion: summed up head rotation in angle degrees, summed up head movement in meters, and total number of teleportation. We further log participants’ interactions: objects created, objects deleted, objects moved, plus every 4 s whether the participant was moving something around in this time span. As with the questionnaires, these data were recorded for each VR exposure.

### Testing procedure

The experiment took approximately 1 h to complete. At first, the study information was read to the participants and they then gave verbal consent. Participants provided demographic background (age, gender, current occupation, VR experience, and medical knowledge) and a baseline FMSS. Next, participants did a short VR training session for around 5 min, to familiarize themselves with the VR application. Afterward, they started with one of the four conditions in a counterbalanced order. Participants worked on a task in the virtual environment for 5 min. The instructions were given within the environment using text displays next to the menu. After the 5 min were over, participants took off the HMD and proceeded to answer the post-experiment questionnaire on a computer. This was repeated four times so that participants experienced both reconstructions in both rooms. After the last condition was completed, they had the option to leave comments on their experience.

### Participants

We recruited 21 participants via mailing lists and word of mouth to take part in the experiment to achieve reasonable statistical power. Of those participants, nine identified as female and twelve as male. The mean age was $M = 24.57$ ($\hbox {SD} = 1.66$) years, all participants were graduate students. Nine participants had little VR experience (less than 5 times overall), nine had some VR experience with occasional use, and three reported high experience with regular use. Prior medical knowledge measured with a 7-point Likert scale *no knowledge (1)* and *detailed knowledge (7)*, resulted in a mean of $M = 2.90$ ($\hbox {SD} = 1.58$). One participant had to be excluded from the objective measures’ data analysis due to technical issues. Participants received no compensation. Exclusion criteria were being underage and severe uncorrected vision.

## Results

We used SPSS version 29 to run our statistical evaluation, calculating two-way repeated measures tests with a significance level of 0.05. While we are mainly interested in the effects of the reconstruction type, we included the room as a factor to be able to evaluate its effects. We did not test for sphericity as both factors have only two levels. We tested all data for normality using the Shapiro–Wilk test, which was either completely normally distributed, or had only minor violations in some scales, unlikely to affect outcomes at our sample size.

### Subjective measures

All main effects for the reconstruction type of the measurements perceived utility, presence, and workload can be found in Table 1.

**Table 1:** Comparisons for perceived utility, IPQ, and NASA-TLX between the different reconstruction types

	Neural reconst.		Whiteboxed		Test statistic	Sign	Effect
	*M*	$\hbox {SE}$	*M*	$\hbox {SE}$	$F(1,20) =$	*p* $\le$	$\eta _{p}^2$
*Perceived utility*							
Confidence	4.89	.173	3.10	.239	60.113	**.001**	.750
Memorability	5.58	.193	4.18	.299	32.599	**.001**	.620
Utility	6.20	.126	4.08	.330	63.793	**.001**	.761
*Presence*							
General	6.19	.088	4.64	.364	17.064	**.001**	.460
Spatial presence	5.93	.079	4.95	.240	17.418	**.001**	.466
Involvement	5.91	.113	4.73	.282	19.764	**.001**	.497
Realism	5.07	.113	2.55	.108	251.709	**.001**	.926
*Task load*							
Mental	47.6	3.994	28.2	3.253	30.249	**.001**	.602
Physical	29.5	5.605	19.5	2.513	9.268	**.006**	.317
Temporal	28.1	4.586	26.2	4.468	.218	.646	.011
Performance	71.5	3.568	72.3	3.370	.047	.830	.002
Effort	42.6	3.641	28.3	3.429	16.889	**.001**	.458
Frustration	18.7	4.044	27.881	4.822	2.976	.100	.130

Descriptive values depict mean (M) and standard error (SE)

#### Perceived utility

We find a significant difference in perceived utility (see “Appendix B”) between the reconstruction types, $F(3,18)=22.661$, $p<.001$, Wilks’$\Lambda =.209$, $\eta _{p}^2=.791$. Effects of the room ($F(3,18)=2.644$, $p<.080$, Wilks’$\Lambda =.694$, $\eta _{p}^2=.306$) and interaction effects ($F(3,18)=1.062$, $p<.390$, Wilks’$\Lambda =.850$, $\eta _{p}^2=.150$) did not reach significant levels. All three subscales were rated significantly higher in the neural reconstruction-based environment. To assess the reliability and internal consistency of our questionnaire, we calculated Cronbach’s alpha. The scale was found to be highly reliable [18] with $\alpha =.929$.

#### Presence

Similarly to utility, we calculate the effects on presence with the four IPQ subscales as input to a MANOVA. There was a significant difference in overall presence between the two visualization types, $F(4, 17)=68.304$, $p<.001$, Wilks’ $\Lambda =.059$, $\eta _{p}^2=.941$. Further, there was no significant effect of the room ($F(4,17)=1.059$, $p<.407$, Wilks’$\Lambda =.801$, $\eta _{p}^2=.199$) and no significant interaction effect ($F(4,17)=1.370$, $p<.286$, Wilks’$\Lambda =.756$, $\eta _{p}^2=.244$). Exploring the four subscales, we find all of them significantly higher in the neural reconstruction-based visualization.

#### Perceived task load

Analyzing the reconstruction type, we find the mental, physical, and effort subscales significantly different between the conditions. The other scales, temporal, performance, and frustration do not differ significantly. Participants generally reported higher workload in the neural reconstruction-based environment compared to the whiteboxed environment, however, with frustration being slightly lower. Additionally, comparing differences between the rooms irrespective of reconstruction type, we find performance ($F(1, 20)=11.310$, $p=.003$, $\eta _{p}^2=.361$ significantly higher in the mock OR ($M=76.1$; $\hbox {SE}=2.729$) compared to the real OR ($M=67.7$; $\hbox {SE}=3.466$).

#### Familiarity with environment

Participants rated their familiarity with the neural reconstructed mock OR $M=5.81$ ($\hbox {SD}=1.08$, with the whiteboxed mock or $M=4.14$ ($\hbox {SD}=1.90$), with the neural reconstructed real OR $M=5.29$ ($\hbox {SD}=1.31$) and with the whiteboxed real OR $M=3.33$ ($\text {SD}=1.88$).

#### Motion sickness

We found no statistically significant difference in reported sickness between our measurement before the experiment started and after each measurement, $F(2.751, 55.024)=0.238$, $p=854$, $\eta _{p}^2=.012$. Reported data are with Greenhouse–Geisser correction applied.

### Objective measures

Tracked participant head movement and rotation both were significantly higher in the reconstructed environment. There was, however, no impact of visualization on teleportation movement actions. It was instead mostly dependent on room size. Table 2 has a detailed listing of objective measures’ effects. We compared participants interactivity as four second time slots spent manipulating objects, as well as sum of all discrete object interaction events. There was no significant difference in participant interactivity between the rooms in either of those cases.

**Table 2:** Objective measures between the two different reconstruction types

	Neural reconst.		Whiteboxed		Test statistic	Sign	Effect
	*M*	$\hbox {SE}$	*M*	$\hbox {SE}$	$F(1,19) =$	$p \le$	$\eta _{p}^2$
Head movement (m)	36.0	2.31	30.3	2.06	6.025	**.024**	.241
Head rotation (^∘^)	7200.8	439.14	5700.5	493.26	21.309	**.001**	.529
Teleportation	7.8	1.33	6.1	0.87	2.449	.134	.114
Interactive time slots	47.6	1.97	42.8	2.85	2.903	.105	.133
Interactions	10.3	0.48	9.2	0.70	2.475	.132	.115

Descriptive values depict mean (M) and standard error (SE)

### Qualitative comments

Even though post-exposure comments were optional, 18 participants positively mentioned the reconstruction quality, real and detail-rich environment of neural reconstruction-based scenarios. Also positively mentioned were the ease of use of the application controls in VR. Negatively mentioned were that the environment sometimes was lacking in detail, not high enough resolution or flat surfaces were not completely flat. Some users further mentioned problems interacting with the menu and objects as desired and difficulties understanding and implementing the tasks.

## Discussion

The perceived usability, including all subscales, was significantly higher in the neural reconstruction-based digital twins. This indicates that this reconstruction type provided users with worthwhile additional details and information to make more informed decisions. Users experienced higher mental and physical workload in the neural digital twins. We expected this to be the case, as there are more details around to process and take in, as indicated by the increased head movement and rotation. While it might be worthwhile to invest in ways to direct users attention to reduce this workload, it is to note that higher workload does not necessarily mean that participants were mentally overloaded, performing worse. Future studies could explore this relationship more. There was no increased interactivity, indicating that the more realistic visuals did not inspire more thorough planning. Together with the increased perceived utility, it stands to reason that the visual detail was responsible for the higher perceived utility, independent of the amount of interaction and planning. Future work could investigate whether hybrid approaches, combining the two visualization strategies, lead to better outcomes. In such cases, lower detail environments could be automatically generated as well, strategically leaving out unimportant details while keeping all relevant information.

TLX performance being significantly higher in the mock OR, compared to the real one, possibly indicates that the tasks were harder there, or participants were less sure about their performance with a real surgical environment. There being no difference in TLX performance between reconstruction types, but in perceived utility, might indicate that novice users did not know how to properly evaluate their planning outcome, but still believe that the visualization would be useful for experts solving clinical planning tasks. Overall increased presence indicates the suitability of the environment, with high realism indicating a high level of detail, and high levels of involvement and spatial presence matching the increased head rotation and users actively exploring and understanding the environment. Analyzing the familiarity values, we see very high averages for the real OR, even though most participants are very unlikely to know the room. The higher realism possibly evokes more memories of other ORs participants have seen, indicating suitability for learning in the right context [19], helping users recall information due to context-based environmental cues. Our approach works well for different environments and has high reconstruction quality, making it worth investigating as a pedagogical tool. These results indicate that future planning tools could invest in enabling users to supply own video material for processing and then planning.

### Limitations

We tested two different VR scenarios focused on being realistic, and easily achievable, thus lacking a comparison to current advanced planning tools. Further, our sample is limited to graduate students. Despite partial medical knowledge, the results cannot be blindly generalized to planning experts. However, we believe that the study gives substantial indications for the feasibility of the approach. Finally, our tasks were fictitious examples and may not resemble realities’ full complexity. Thus, some of our findings might not generalize fully beyond the specific scenarios of this study. Further studies should therefore substantiate the results of the present investigation, possibly utilizing clinical professionals, real planning or emergency tasks and an expanded set of environments and objects. Integration into simulation environments could help quantify planning success in addition to field tests. Discrete event simulation was shown to be able to measure and compare virtual ORs along multiple axes like spatial or hygienic requirements [20] and could potentially also integrate analyses directly into the virtual environment.

### Conclusion

Our results indicate suitability of neural reconstructed digital twins for medical planning tasks. Utilizing such visualizations increased perceived utility and presence compared to a low visual fidelity environment. Moreover, we show that current neural reconstructions can be created and used with off-the-shelf hardware, providing real-world benefits.

### Supplementary Information

Below is the link to the electronic supplementary material.

Supplementary file 1 (mp4 69243 KB)

## Figures and Tables

**Fig. 4:**
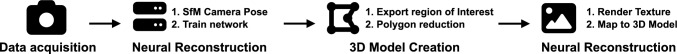
Reconstruction pipeline. Data are acquired with a phone or camera, poses for each picture are determined by a structure from motion algorithm, a reconstruction is trained with the located images, a mesh is exported by running marching cubes on the signed distance field and then decimated, a texture is rendered for mapped to the exported mesh

**Fig. 5:**
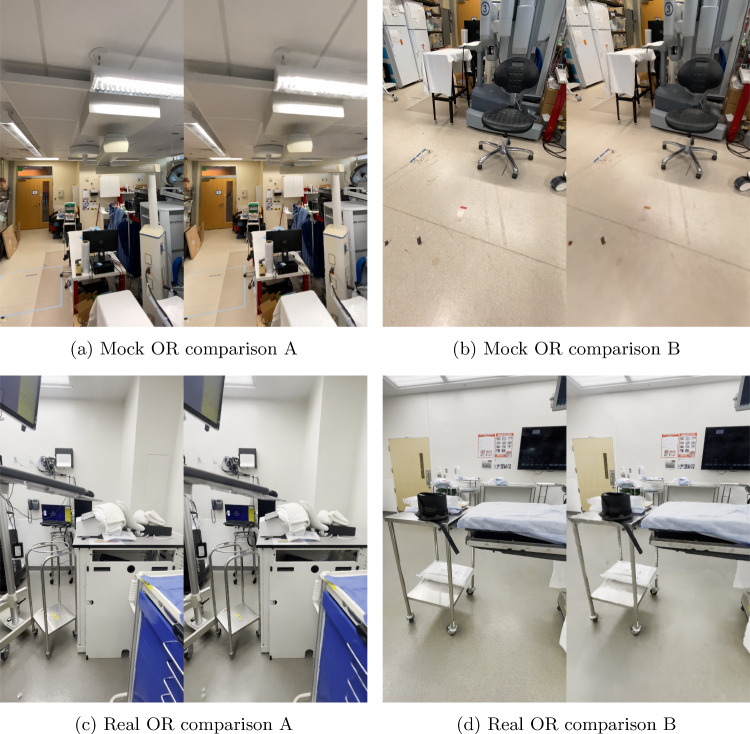
Comparison images highlighting the reconstruction quality of the used environments. Input photographs are on the left and reconstructions are on the right in each comparison

**Fig. 6:**
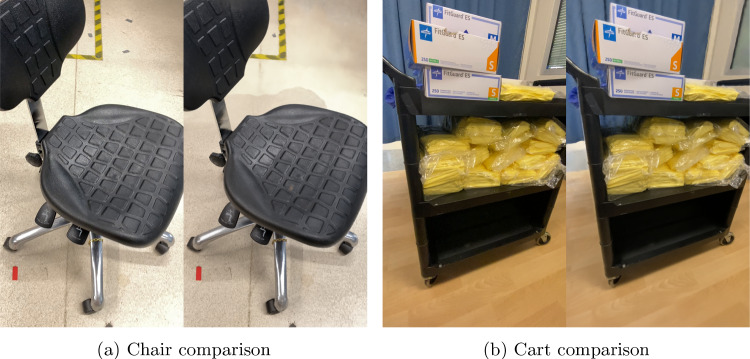
Comparison images highlighting the reconstruction quality of the used objects. Input photographs are on the left and reconstructions are on the right in each comparison

## Appendix A: Tasks

Tasks participants had to work on in the virtual environments.

### Mock OR

- Task: Update Hardware! Needed: a surgical robot, one or more tracking carts, a TV cart, space for at least two surgeons. Goals: Remove old tripods and place new tracking carts with clean line of view of the patient. Surgeons can easily manipulate the robot and see the monitor. Robot can reach the patient.
- Task: Prepare long procedure! Needed: Sterile cart, trolley, two or more surgeons, chairs and place to lay down tools. Goal: Surgeons should be able to move freely, while being able to easily access tools in the trolley and lay them on the table. Chairs should be accessible as needed to sit down without obstructing paths.

### Real OR

- Task: Plan Surgery! Needed: a surgical robot, a TV cart, tracking trolley, table and space for at least two surgeons. Goal: Surgeons can easily manipulate the robot and reach patient, table and trolley. The TV should be well visible, but not in the way.
- Task: Transfer Patient! Needed: a surgical robot, a tracking tripod, a monitor and space for patient table, sterile material cart and medical professionals. Goal: Make sure a patient bed can move right next to operating table. Find the best layout to transfer patient, then back to surgery easily, while having access to sterile material.

## Appendix B: Perceived utility instrument

See the Table 3.

**Table 3:** Custom perceived utility scale used in our experiment, rated on a 7-point Likert scale

Nos	Subscale	Question
1	Confidence	I am confident my planning is suitable for usage in a real-world scenario
2	Confidence	I feel there could be unforeseen problems with my planned design
3	Memorability	I understood the layout of the virtual room
4	Memorability	I can easily remember what the virtual environment was like
5	Memorability	I feel like I could accurately recreate the setup in the real room
6	Utility	I felt the visualization helped me successfully plan the new room layout
7	Utility	I think the system could be used to plan real operating rooms
8	Utility	I think the visualization was adequate to achieve satisfactory results

## Appendix C: Reproduction information

We used a workstation with the following hardware for Neuralangelo training:

- AMD Ryzen 9 3950X 16-Core Processor
- Two NVIDIA Quadro RTX 6000 24 GB memory graphics cards
- 128 GB RAM
- Operating system: Ubuntu 20.04
- NVIDIA driver version 470.182.03
- CUDA version 11.4

The rooms were reconstructed using both GPUs. Objects were reconstructed using a single GPU and a reduced hash map size of $2^{21}$. All other parameters were as described in [14]. While we used a custom implementation, open-source implementations of the Neuralangelo reconstruction approach are available from Nvidia (https://github.com/NVlabs/neuralangelo) and as part of SDFStudio (https://github.com/autonomousvision/sdfstudio).

The input data were collected with recent model Apple iPhones (11 and 14), with the built-in wide-angle lens and high framerate to reduce motion blur artifacts. The reconstruction pipeline is also shown in Fig. 4.

The study supervisor monitored the experiment using a 15-inch Razer Blade laptop. Participants used the same machine to fill out the questionnaires after the VR exposure.

## Appendix D: Reconstruction quality comparison

This section contains exemplary comparison images to judge the neural reconstruction output before SDF to mesh conversion or texture mapping has been applied. Raw camera input data are on the left of each image and neural reconstruction output on the right. Figure 5 highlights reconstructions of the environments, Fig. 6 displays comparisons of the objects.
